# The value of diagnostic experience in O-RADS MRI score for ovarian-adnexal lesions

**DOI:** 10.1515/med-2025-1316

**Published:** 2025-12-17

**Authors:** Miao Jin, Tingting Huang, Dongxue Wang, Hongsheng Cui, Xiaomeng Xu

**Affiliations:** Department of Magnetic Resonance, The Third Affiliated Hospital of Qiqihar Medical University, Qiqihar City, China; Department of Gynaecology and Obstetrics, The Third Affiliated Hospital of Qiqihar Medical University, Qiqihar City, China

**Keywords:** diagnostic experience, ovary-adnexa, tumor, O-RADS, magnetic resonance imaging, score

## Abstract

**Objectives:**

To evaluate the impact of radiologists’ experience on the diagnostic accuracy of O-RADS MRI risk stratification system for ovarian-adnexal lesions.

**Methods:**

This single-institute retrospective study include 252 lesions from 240 cases. Two experienced (Group A) and two inexperienced radiologists (Group B) independently assessed lesions using O-RADS MRI risk stratification system. Inter-reader agreement and diagnostic performance were analyzed using Kappa statistics and chi-square tests.

**Results:**

The O-RADS MRI showed significant consistency within and between groups (Kappa=0.879, p<0.001 for Group A; Kappa=0.469, p<0.001 for Group B; Kappa=0.695, p<0.001 for inter-group). Group A outperformed Group B in specificity, accuracy, and positive predictive value. For O-RADS MRI scores 3–5, Group A demonstrated higher accuracy in qualitative diagnosis, particularly for score 3 (96.77 % vs. 57.69 %). The sensitivity of scoring classification was consistently higher in Group A across all scores.

**Conclusions:**

Extensive diagnostic experience significantly enhances the diagnostic performance of the O-RADS MRI risk stratification system. A more refined O-RADS MRI risk stratification system, a typical case image library and AI-assisted interpretation hold promise for narrowing the diagnostic disparities between experienced and inexperienced readers.

## Introduction

Ovarian-adnexal lesions are common in the female reproductive system, affecting individuals across various age groups, including children and those of childbearing age [1], [2], [3]. Benign ovarian lesions (such as functional cysts, endometriomas, and benign neoplasms) are far more prevalent than malignant ones, with study reporting that up to 69 % of ovarian masses are benign and mainly in premenopausal women [4], 5]. However, ovarian cancer remains the most lethal gynecologic malignancy, with global statistics from 2020 showing an incidence rate of 6.6 % and a mortality rate of 4.2 %, while the 5-year survival rate for advanced-stage patients is below 50 % [6], [7], [8]. Accurate diagnosis of these lesions is crucial to avoid misdiagnosis of malignant tumors and unnecessary reproductive function loss due to overtreatment of benign conditions [2], 9]. However, the complexity of ovarian-adnexal lesions, which can include tumor-like lesions, epithelial tumors, sex cord-stromal tumors, germ cell tumors, metastases, and collision tumors, poses diagnostic challenges. These lesions may contain diverse components such as serous fluid, mucus, fat, blood, clots, calcifications, and fibrous tissue, further complicating accurate assessment [10], [11], [12].

Ultrasound remains the first-line imaging modality due to its wide availability, low cost, and real-time dynamic assessment capability. Transvaginal ultrasound improves resolution for detecting small lesions and assessing vascularity via Doppler imaging. However, its limitations include operator dependency and reduced sensitivity (18–31 % of lesions may be missed), particularly in obese patients or those with complex pelvic anatomy [1], 13]. Computed tomography is primarily used for staging malignant lesions, detecting lymph node involvement, and identifying distant metastases. However, it has limited utility in characterizing indeterminate adnexal masses due to poor soft-tissue contrast [14], 15]. Magnetic Resonance Imaging (MRI) with its superior soft-tissue resolution and multi-parametric imaging capabilities, provides a more comprehensive approach to localizing and characterizing pelvic lesions, serving as a vital supplementary diagnostic tool [16], [17], [18], [19]. However, it remains limited by significant subjectivity and the inability to provide quantitative malignancy risk assessments. The application of artificial intelligence (AI) in adnexal lesion diagnosis is becoming increasingly widespread. Using deep learning and machine learning, AI enhances the diagnostic accuracy for ovarian-adnexal lesions and achieves high Area Under the Curve (AUC) values in clinical studies. However, this technology still faces challenges, including generalizability across different imaging protocols, ethical concerns in AI-driven decision-making, and the need for prospective validation in clinical practice [20], 21]. In 2022, the American College of Radiology introduced the Ovarian-Adnexal Reporting and Data System Magnetic Resonance Imaging (O-RADS MRI) risk stratification system [22]. Although the O-RADS MRI risk stratification system has demonstrated high sensitivity and specificity in assessing malignant risk, most studies have involved experienced radiologists, leaving a gap in understanding its applicability to less experienced practitioners [23], [24], [25]. This study aims to evaluate the impact of diagnostic experience on O-RADS MRI score and identify key factors influencing its accuracy by comparing the consistency and diagnostic efficacy between experienced and inexperienced radiologists.

## Methodology

### Patients

This single-institute study was approved by the ethics committee of Qiqihar Medical University (Approval ID: QMU Ethical Review [2023] No. 38). Informed consent was obtained from all patients or their families. We enrolled 240 patients with 252 ovarian-adnexal lesions, all confirmed by pathological examination at Qiqihar Medical University’s Pathological Examination Center. All patients underwent surgery at the Third Affiliated Hospital of Qiqihar Medical University, Department of gynaecology and obstetrics between June 2018 and January 2025. Inclusion criteria: a) Successful pelvic MRI examination performed according to O-RADS MRI scanning protocol. b) Presence of an ovarian-adnexal lesion. c) No prior treatment before MRI examination. d) Availability of pathological results. Exclusion criteria: a) Non-diagnostic-quality imaging. b) Suspected ectopic pregnancy. c) Maximum lesion diameter <5 mm (Table 1).

**Table 1:** Inclusion and exclusion criteria.

Category	Criteria
Inclusion criteria	a) Successful pelvic MRI examination performed according to O-RADS MRI scanning protocol
	b) Presence of an ovarian-adnexal lesion
	c) No prior treatment before MRI examination
	d) Availability of pathological results
Exclusion criteria	a) Non-diagnostic-quality imaging
	b) Suspected ectopic pregnancy
	c) Maximum lesion diameter less than 5 mm

### Equipment and methods

After adequate pre-examination preparation, patients were positioned on the scanner table [26]. Pelvic MRI scans were performed using a GE Discovery 3.0T scanner with an abdominal coil. The scan range extended from the anterior superior iliac spine to the symphysis pubis, ensuring full coverage of large lesions. Dynamic contrast-enhanced (DCE) scanning began 30 s before contrast agent injection to acquire a mask image. Continuous scanning was performed to obtain DCE images, with a total imaging duration of 1 min and a temporal resolution of ≤15 s [27]. A high-pressure syringe (CM-60, Antmed Medical Technology Co., Ltd.) was used to administer the contrast agent (gadoterate meglumine, 0.2 mL/kg body weight), followed by a 15 mL saline flush, both injected at a rate of 2 mL/s. For non-dynamic enhanced scanning, enhanced images were acquired 30–40 s after contrast injection. Detailed scan sequences and parameters are provided in Table 2.

**Table 2:** MRI scan sequences and parameters.

Scanning sequence	TR, ms	TE, ms	FOV, mm × mm	Thickness, mm	Intersection gap, mm	Matrix
Axial T1WI	821	10	280 × 280	5	1	352 × 352
Sagittal T2WI	1,703	68	340 × 340	5	1	352 × 352
Axial T2WI-Fs	12,414	91	280 × 280	5	1	352 × 352
Coronal T2WI	1,703	66	450 × 450	3	1	352 × 352
DWI b1000DWI b1000	2,000	50	300 × 200	3	1	352 × 352
Axial WATER: LAVA-Flex	3.6	1.7	480 × 430	3	1	352 × 352

Fs, fat saturation sequence; WATER, LAVA-Flex, enhanced sequence; LAVA, three-dimensional liver accelerated volume acquisition imaging; TR, repetition time; TE, echo time; FOV, field of view.

### Image analysis and post-processing

a) Measurement of maximum lesion diameter. b) Assessment of T2-weighted image (T2WI) and Diffusion weighted imaging (DWI) signal characteristics using the iliopsoas muscle as reference. c) Documentation of loculus number. d) Generation of time-intensity curves (TICs) for DCE scans, with the lesion region of interest (ROI) placed in the earliest enhancing solid area and myometrial ROI in vessel-free regions; For non-DCE cases, lesions with enhancing solid tissue (excluding T2-dark/DWI-dark lesions) were evaluated against myometrium at 30–40 s post-injection. e) O-RADS MRI score based on established criteria [22].

Radiologists with 10–12 years’ experience (Group A: A1 and A2) and 2 years’ experience (Group B: B1 and B2) independently characterised adnexal lesions according to a standardised lexicon without knowledge of the pathological results and each lesion received an O-RADS MRI score. Group results represented consensus readings. Bilateral lesions were assessed separately.

### Statistical analysis

IBM SPSS 27.0 was used for statistical analyses, with significance set at p<0.05 (two-sided). First, the Kappa test was used to analyze the interobserver agreement of O-RADS MRI score between radiologists A1 and A2, radiologists B1 and B2, and group A and B. Then, lesions were classified as benign or malignant (borderline tumors categorized as malignant) for chi-square tests comparing the overall diagnostic performance – including sensitivity, specificity, positive predictive value (PPV), negative predictive value (NPV), and accuracy – as well as the accuracy of each O-RADS MRI score between groups. The chi-square tests were conducted under the following conditions: all cells had expected frequencies (E) of at least 5. If the number of cells with 1≤E<5 was lower than 20 %, Fisher’s exact test was used. If there were any cells with E<1 or more than 20 % of cells had E<5, statistical values were not recorded. Last, calculated scoring classification sensitivity (correctly assigned scores/total cases) of each O-RADS MRI score for both groups.

## Results

A total of 252 lesions (204 benign, 48 malignant) were enrolled (Table 3). The distribution of lesions by O-RADS MRI score was as follows: 3 lesions scored 1, 113 lesions scored 2, 91 lesions scored 3, 27 lesions scored 4, and 18 lesions scored 5 (Figures 1–5). Morphological analysis identified 45 unilocular simple cysts, 55 unilocular complex cysts, 34 multilocular simple cysts, 53 multilocular complex cysts, and 65 solid-containing lesions. Maximum lesion diameter ranged from 15 to 283 mm (mean: 90 mm). 29 cases underwent dynamic enhanced MRI scan, 24 cases underwent non-dynamic enhanced MRI scan, and 187 cases without enhanced MRI scan [24]. Patient age range was 8–78 years (mean: 46.6 years).

**Table 3:** General characteristics of patients.

	Number	%
Benign (204)		
Ovarian cyst with simple fluid	19	7.54
Ovarian cyst with hemorrhage	11	4.37
Pyosaipinx	5	1.98
Endometrioma	53	21.03
Fibroma necrosis mixed with bleeding	1	0.40
Fibrothecoma	5	1.98
Cystadenofbroma	6	2.38
Serous cystadenoma	60	23.81
Mucinous cystadenoma	20	7.94
Seroucinous cystadenoma	8	3.17
Mature teratoma	16	6.35
Borderline (13)		
Serous borderline tumour	10	3.97
Mucinous borderline tumour	3	1.19
Malignant (35)		
Serous carcinoma	30	11.91
Metastasis	2	0.79
Clear cell carcinoma	3	1.19

**Figure 1:**
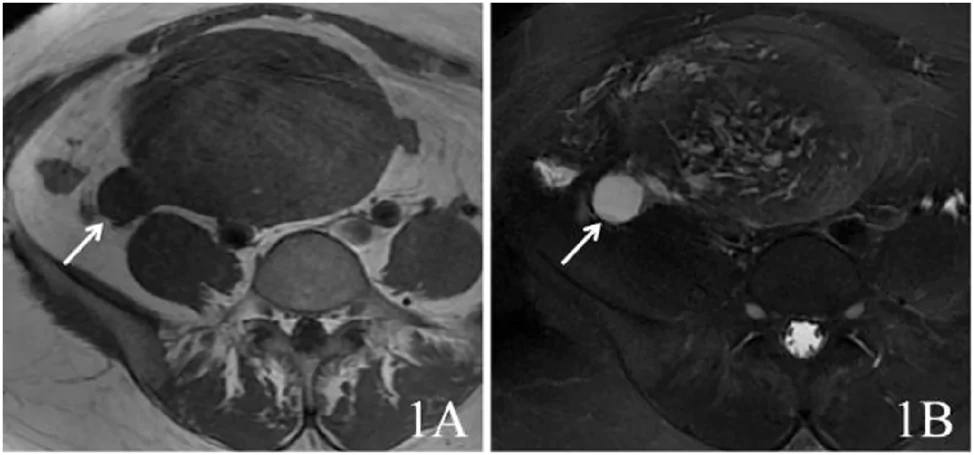
A-B female, 41 years old, corpus luteum cyst, O-RADS MRI 1. A. T1-weighted image (T1WI) revealed a round hypointense lesion (→) in the right pelvic cavity. B. The lesion appears as uniformly high signal intensity on T2-weighted fat-suppressed image (T2WI-FS).

**Figure 2:**
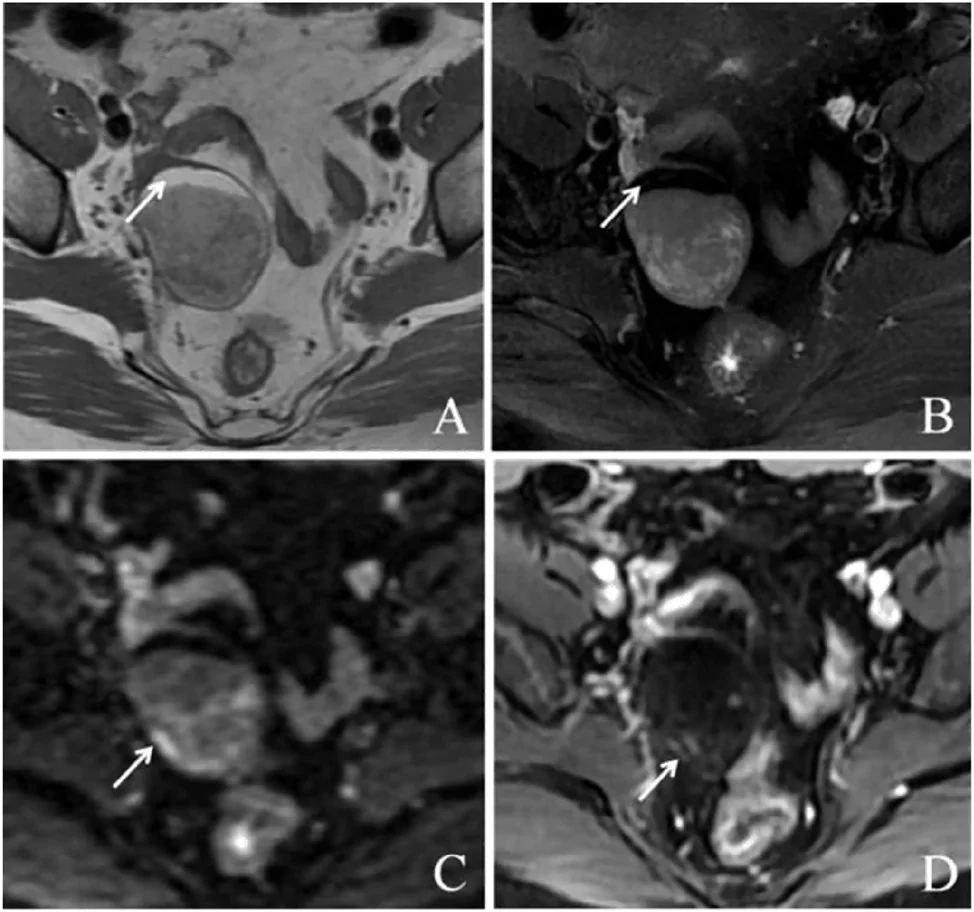
A-D female, 50 years old, mature teratoma, O-RADS MRI 2. A. T1WI revealed an elliptical mass in the right pelvic cavity. The anterior portion shows high signal intensity (→), while the posterior portion exhibited heterogeneous signal intensity. B. T2WI-FS demonstrated hypointensity in the anterior part (→) and heterogeneous hyperintensity in the posterior part. C. Diffusion weighted imaging (DWI) revealed patchy mild hyperintensity in the posterior portion (→). D. Contrast-enhanced imaging showed focal nodular enhancement in the posterior part of the lesion (→).

**Figure 3:**
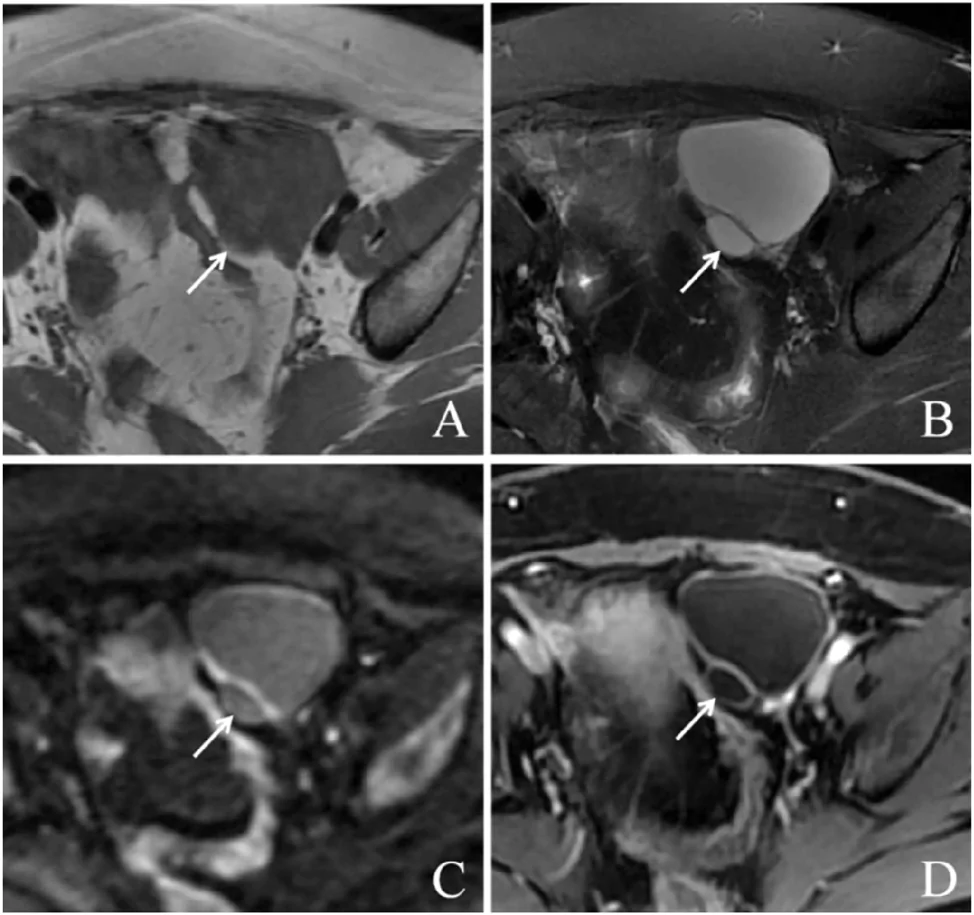
A-D female, 38 years old, serous cystadenoma, O-RADS MRI 3. A. T1WI revealed an irregular hypointense lesion (→) in the left side of the pelvic cavity. B. T2WI-fs demonstrated a uniformly high signal lesion with a septation. C. DWI showed hyperintensity within the septations. D. The post-contrast scan revealed enhancement of the cystic wall without enhancement of the cystic cavity.

**Figure 4:**
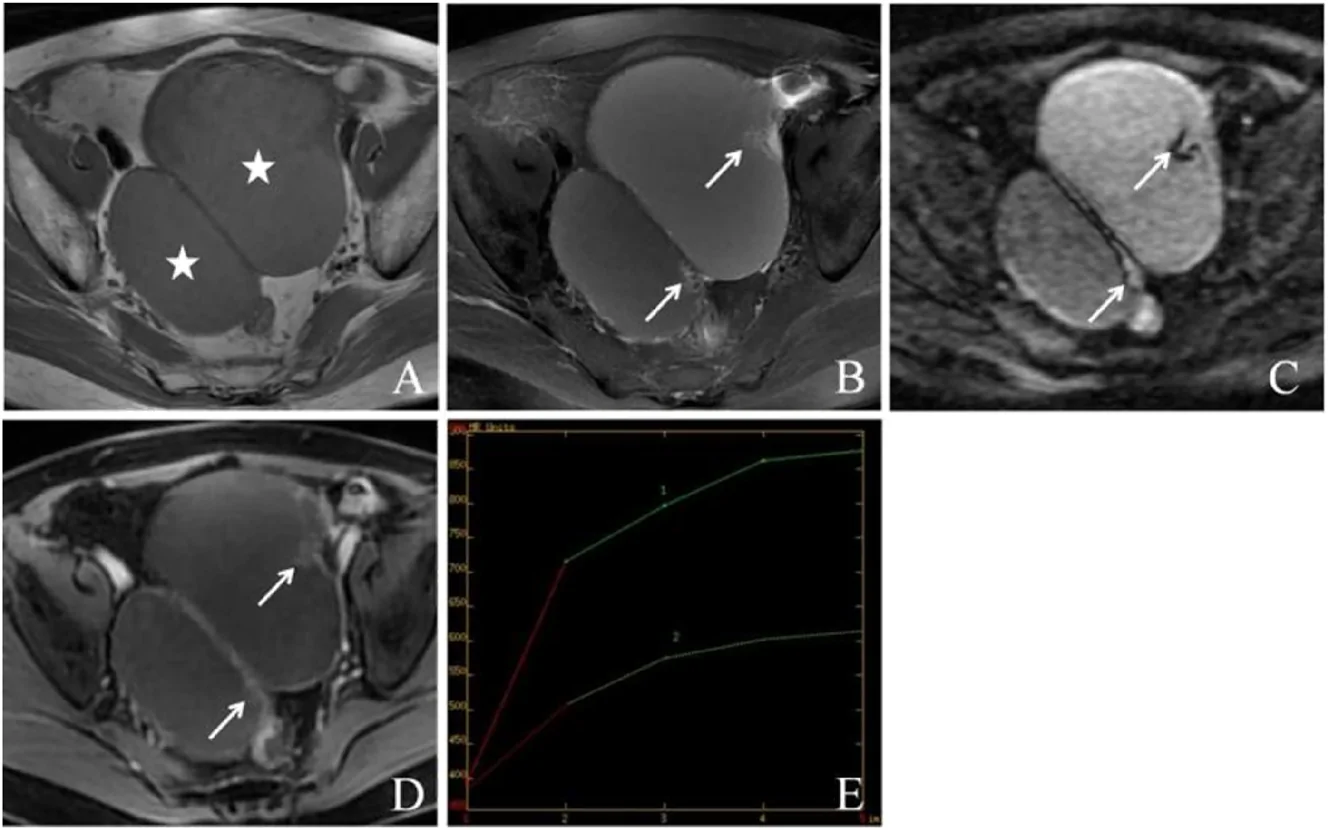
A-E female, 68 years old, borderline serous cystadenomas, O-RADS MRI 4. A. T1WI showed two elliptical hypointense lesions (☆) in the pelvic cavity. B. T2WI-fs revealed low signal lesion with clustered high signal (→) beneath the cystic wall. C. On DWI, the clustered structure showed slightly high signal (→). D.The enhanced scan demonstrated enhancement of the clustered structure (→) beneath the cystic wall, while the cystic cavity remained non-enhanced. E. The TIC showed that the clustered lesion (curve 2) exhibits a slower initial enhancement rate than the uterine myometrium (curve 1).

**Figure 5:**
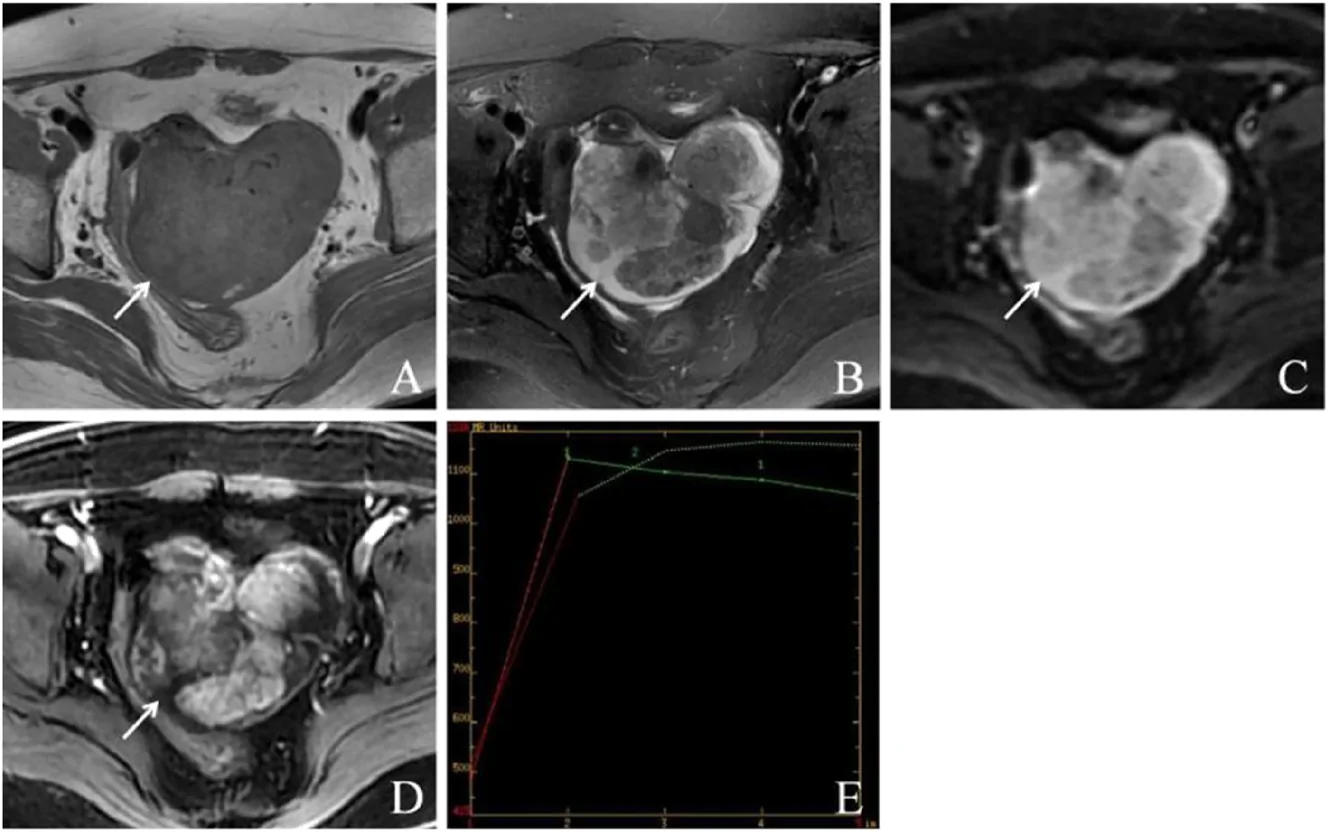
A-E female, 59 years old, serous cystadenocarcinoma, O-RADS MRI 5. A. T1WI showed an irregular hypointense lesion (→) in the pelvic. B. On T2WI-fs, the lesion showed heterogeneous signal intensity (→). C. DWI showed high signal within the lesion (→). D.The enhanced scan demonstrated enhancement of the solid components of the lesion (→). E. The TIC showed that the lesion (curve 2) exhibited a faster initial enhancement rate than the uterine myometrium (curve 1).

Diagnostic result of Groups A and B were summarized in Table 4. O-RADS MRI showed strong interobserver agreement (Group A Kappa=0.879, Group B Kappa=0.469, inter-group Kappa=0.695; all p<0.001) (Table 5). Group A showed superior diagnostic performance, with significantly higher specificity (94.12 % vs. 73.53 %; p<0.001), accuracy (94.05 % vs. 76.19 %; p<0.001), and PPV (78.95 % vs. 43.75 %; p<0.001) compared to Group B (Table 6). For O-RADS MRI scores 3–5, Group A demonstrated higher accuracy in qualitative diagnosis, particularly for score 3 (Table 7). The sensitivity of scoring classification was consistently higher in Group A compared with Group B across all O-RADS scores (score 1: 66.67 % vs. 33.33 %; score 2: 84.07 % vs. 82.30 %; score 3: 91.21 vs. 76.92 %; score 4: 100 % vs. 88.89 %; score 5: 100 % vs. 83.33 %).

**Table 4:** The O-RADS MRI score result of group A and B (number).

Group A	Group B						
	Score 1	Score 2	Score 3	Score 4	Score 5	Total	Malignant
Score 1	0	3	0	0	0	3	0
Score 2	6	84	0	6	0	96	0
Score 3	0	9	69	12	3	93	3
Score 4	0	0	6	30	3	39	27
Score 5	0	0	3	3	15	21	18
Total	6	96	78	51	21	252	
Malignant	0	0	9	24	15		48

**Table 5:** Intra-group and inter-groups consistent of O-RADS MRI risk stratification system.

	Kappa value	p-Value
Radiologists A1-A2	0.879	0.001
Radiologists B1-B2	0.469	0.001
Group A-B	0.695	0.001

**Table 6:** The overall qualitative diagnostic efficacy of group A and Group B (%).

	Group A	Group B	χ2 value	p-Value
Sensitivity	93.75	87.5	–	–
Specificity	94.12	73.53	31.885	0.001
Accuracy	94.05	76.19	31.720	0.001
PPV	78.95	43.75	18.063	0.001
NPV	98.46	96.15	–	–

**Table 7:** The qualitative diagnostic accuracy of each score of group A and Group B (%).

	Group A	Group B	χ2 value	p-Value
Score 1	100	100	–	–
Score 2	100	100	–	–
Score 3	96.77	57.69	38.984	0.001
Score 4	76.92	58.82	3.258	0.071
Score 5	85.71	71.43	1.273	0.259

## Discussion

Our findings indicate that the O-RADS MRI risk stratification system demonstrates consistency among both experienced and inexperienced radiologists, as well as between the two groups. This supports the notion that the O-RADS MRI risk stratification system is a widely applicable diagnostic tool, consistent with previous reports [28], 29]. However, the consistency between experienced radiologists (Kappa=0.879) was higher than that between inexperienced radiologists (Kappa=0.469) and between the two groups (Kappa=0.695). This aligns with a previous study (Kappa=0.43) involving readers with 2 years of experience [30], suggesting that experienced radiologists are more consistent in recognizing and interpreting lesion features, while inexperienced radiologists are more prone to discrepancies or errors. Furthermore, the sensitivity of scoring classification for each score was higher in Group A than in Group B. This discrepancy may be attributed to several challenges faced by inexperienced radiologists: 1) Difficulty in differentiating certain cystic lesions, such as simple ovarian cysts from mesenteric cysts and hemorrhagic ovarian cysts from endometriotic cysts [31], 32]. Additionally, some multiple cystic lesions were misdiagnosed as a single multilocular lesion. For example, endometriosis may present with several smaller cysts (<1 cm) surrounding a larger cyst on T1 weighted image (T1WI) [33], 34], which were referred to as “satellite sacs” (Figure 6), this feature may confuse inexperienced radiologists. We propose that “satellite sacs” should be scored as 2 and not mistaken for multilocular lesion and assigned a higher score. 2) Different interpretations of deep lobulated cystic lesions and multilocular cystic lesions led to scoring errors. We suggest that when the communication diameter of cysts exceeds half the short diameter of the smaller cyst, it should be considered a deep lobulated lesion (Figure 7). 3) Misjudgment of teratomas with minimal fat content or those resembling pelvic fat as non-teratomas. Additionally, the guidelines classify lesions with lipid and large-volume enhanced solid tissue as score 4. Inexperienced readers’ varying interpretations of “large volume” led to scoring errors [35], 36]. To address this, we propose defining “large volume” as greater than 1/3 of the lesion volume, based on our observations in 40 cases of mature teratoma and 2 immature teratoma. However, this requires further validation in a larger sample of enhanced scanning cases.

**Figure 6:**
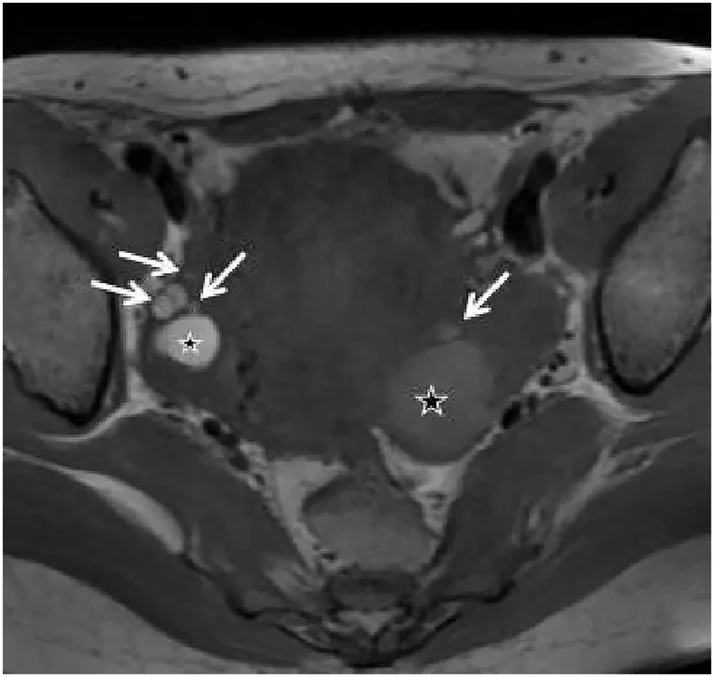
Female, 42 years old, endometrioma. T1WI showed several smaller cysts (→) surrounding a larger cyst (☆) in both ovaries, which are referred to as “satellite sacs”.

**Figure 7:**
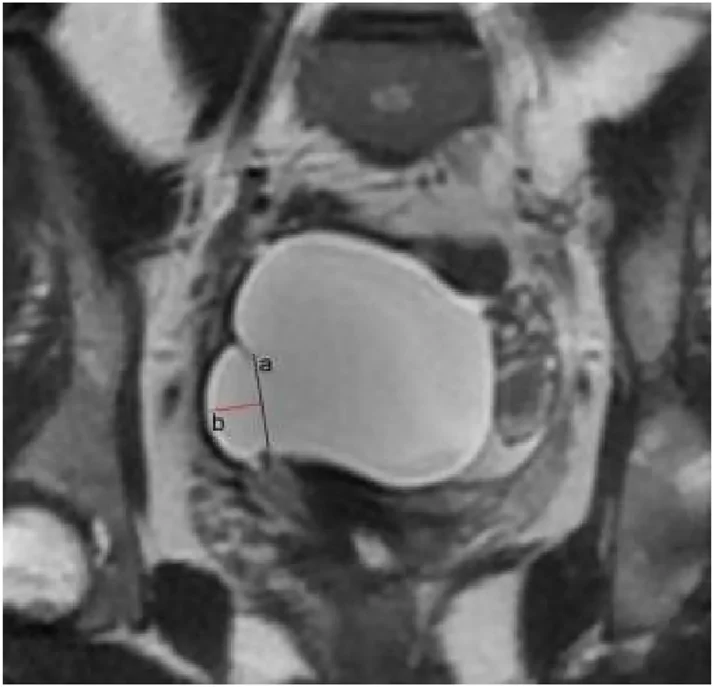
Female, 23 years old, serous cystadenoma. On T2WI, the measurement of “a” was greater than half of “b” (a>b/2), which should be considered a deeply lobulated cystic lesion.

In our study, compared to Group A, Group B had fewer cases with score 3 but a higher proportion of malignancies among them, and more cases with score 4 but a lower proportion of malignancies. Group B’s higher malignant proportion in score 3 stemmed from inaccurate identification of solid components. Non-solid elements – including overlapping septations in cystadenomas (Figure 8), thickened adhesions around endometriotic cysts, retracted ovarian parenchyma (Figure 9), and tubo-ovarian abscess walls – were sometimes misidentified as solid components. Undetected small papillary structures also led to borderline lesions being under-scored as 3, imaging diagnosis of borderline cystadenomas remains challenging, particularly for less experienced physicians. The literature indicates that the presence of papillary architecture with internal branching (PA&IB) is a characteristic MRI finding for serous borderline ovarian tumors (SBOTs), yet it appears in only 69.7 % of cases. Moreover, MRI can not differentiate subtypes (SBOT-MP vs. SBOT-C). Histopathology remains the gold standard for diagnosing borderline cystadenomas, though micro-papillary patterns may be occult and require expert pathological review. Consequently, identifying borderline cystadenomas necessitates a multimodal assessment. Advances in molecular subtyping technologies show promise for further enhancing diagnostic accuracy [37], [38], [39]. In a word, accurate identification of solid components is crucial for scoring, which consistent with literature reports [32], 40]. Group A demonstrated superior diagnostic efficiency, attributable not only to solid component recognition but also to better understanding of enhancement characteristics. For example, Group B misclassified a fibrothecoma as malignant (score 4) because its atypical presentation (absent “dark T2/dark DWI” signals; early non-dynamic enhancement lower than myometrium) wasn’t accommodated by score 3 guidelines. Literature additionally documents misinterpretations of DCE curves, where insufficient delay time may cause medium-risk lesions to be misclassified as low-risk due to obscured shoulders or plateaus – though not observed in our study, this warrants vigilance [29], 32], 41]. Furthermore, ovarian pedicle torsion can reduce enhancement (due to compromised blood supply), risking false-negatives [29], 40], 42]. In our study, Group B failed to identify a case of teratoma with pedicle torsion. Fortunately, this oversight did not lead to a diagnostic error. However, accurately identifying torsion is crucial for lesion classification, and this incident raises questions about the applicability of the risk stratification system in torsion cases.

**Figure 8:**
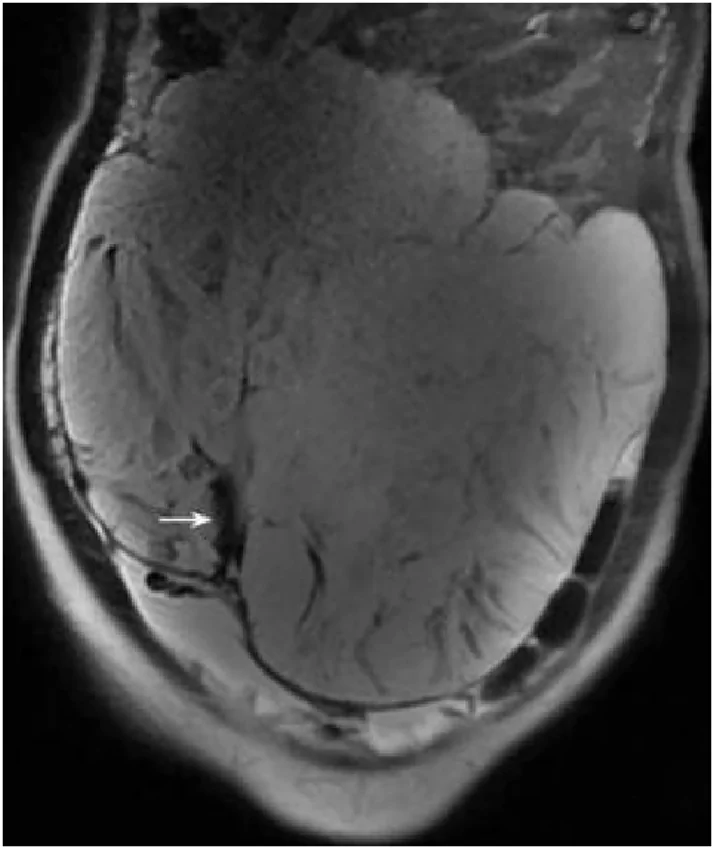
Female, 54 years old, mucinous cystadenoma. T2WI revealed multiple overlapping septations (→) that resemble solid tissue in appearance.

**Figure 9:**
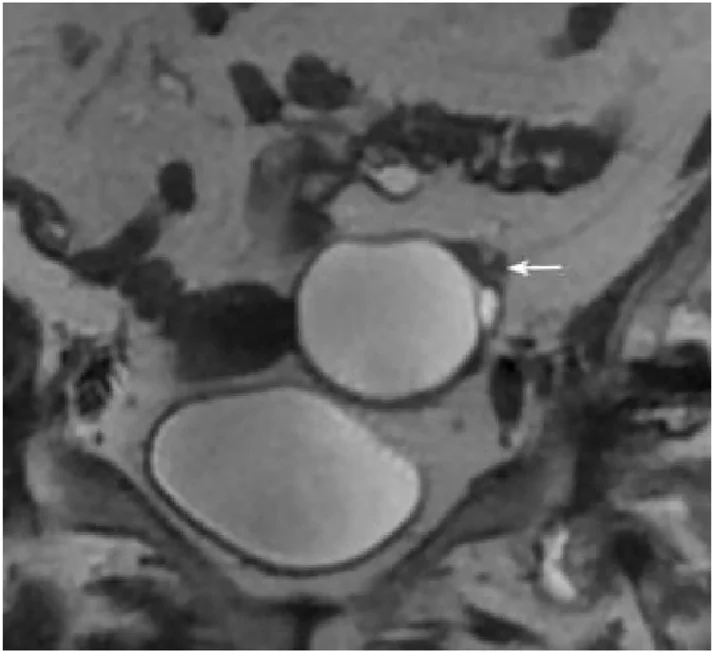
Female, 53years old, serous cystadenoma. T2WI showed the ovarian parenchyma (→) which similar to solid tissue.

In the imaging diagnosis of ovarian adnexal lesions, ultrasound demonstrates lower specificity compared to MRI, with values ranging only from 76 % to 86 % [43]. Previous studies have also shown that MRI achieves higher diagnostic accuracy than ultrasound in distinguishing benign from malignant lesions [23], 44]. However, all these assessments were conducted by experienced radiologists. Our study reveals that inexperienced radiologists achieve suboptimal performance in the using of O-RADS MRI risk stratification system, achieving a sensitivity of only 87.5 % and specificity of only 73.53 %. Therefore, it is necessary to enhance the O-RADS MRI risk stratification system diagnosis performance for inexperienced radiologists. Based on our analysis, we believe that refining the scoring criteria and establishing a typical case image library will help improve the diagnostic performance of ORADS MRI risk stratification system for radiologists with less experience.

In addition, recent rapid advancements in AI have enabled less experienced sonographers to achieve “instant expertise”: A study demonstrated that AI’s diagnostic performance in distinguishing benign from malignant ovarian tumors matched that of experts. In a large-scale study spanning 20 medical centers, AI assistance improved non-expert examiners’ F1 scores from 74.1 % to 82.7 %, surpassing experts’ 79.5 %. Several MRI-based artificial intelligence studies have shown that AI’s diagnostic performance for prostate diseases or qualitative diagnosis of soft tissue tumors matches that of radiology experts, with diagnostic speed reaching up to 400 times faster than human experts. AI outline a replicable “upgrade path” for less experienced MRI radiologists – rapidly narrowing the experience gap to reduce diagnostic dilemmas caused by lack of experience. However, AI is not infallible. Current limitations include device variability, scarcity of rare pathological samples, and lack of clinical information, making false negatives unavoidable. Regulatory frameworks have also not fully clarified liability for AI-related missed diagnoses. Therefore, future efforts should continuously expand multi-brand device datasets, incorporate multimodal clinical data, conduct prospective trials, and ultimately ensure less experienced radiologists safely benefit from this technology [17], [45], [46], [47], [48], [49].

## Conclusions

The O-RADS MRI risk stratification system is a widely applicable and effective diagnostic tool. However, extensive diagnostic experience can significantly enhance the diagnostic performance. Our findings also indicate that a more refined O-RADS MRI risk stratification system and a typical case image library will help improve the diagnostic performance for less experienced radiologists. Additionally, AI-assisted interpretation holds promise for narrowing the diagnostic disparities between experienced and inexperienced readers.

### Limitations

The limitations of this study include the small sample size of dynamic enhancement scans and the limited variety of lesion types. Therefore, further work is to expand the sample size of cases with dynamic enhancement scans and include a greater variety of lesion types to conduct more in-depth research on the factors influencing the accuracy of the risk stratification system.
